# Machine learning-driven multi-omics integration of urinary organic acids and ions enables precision risk stratification for calcium oxalate nephrolithiasis

**DOI:** 10.3389/fmed.2026.1808076

**Published:** 2026-04-20

**Authors:** Peizhi Zhang, Yang Liu, Boxing Su, Yingkun Xu, Zheng Xu, Tianxiang Zhang, Yuxian Wang, Jiangtao Yang, Yawei Liu, Jianxing Li

**Affiliations:** 1 Department of Urology, Beijing Tsinghua Changgung Hospital, School of Clinical Medicine, Tsinghua University, Beijing, China; 2 School of Clinical Medicine, Tsinghua University, Beijing, China; 3 Department of General Surgery, Qilu Hospital of Shandong University, Jinan, Shandong, China; 4 Shenzhen Aone Medical Laboratory Co., Ltd., Shenzhen, China; 5 Health Service Department of the Guard Bureau of the Joint Staff Department, Beijing, China

**Keywords:** calcium oxalate nephrolithiasis, gas chromatography-mass spectrometry, machine learning, metabolic biomarkers, urinary ions, urinary organic acids

## Abstract

**Background:**

Calcium oxalate (CaOx) nephrolithiasis is closely associated with metabolic dysregulation, while current risk assessment based on 24-h urine analysis is time-consuming and inconvenient. This study aimed to develop a noninvasive predictive model for CaOx stones using morning urine organic acid and inorganic ion profiles combined with machine learning, and to identify metabolomic biomarkers related to CaOx stone formation.

**Methods:**

A total of 232 CaOx stone formers and 238 healthy controls were enrolled. Organic acids and inorganic ions in morning urine were quantified by gas chromatography–mass spectrometry and ion chromatography, respectively. Participants were randomly divided into training and testing sets (8:2). Diagnostic models were constructed using random forest, support vector machine, logistic regression, and extreme gradient boosting, with 10-fold cross-validation for optimization. Model performance was evaluated using AUC, accuracy, sensitivity, specificity, F1-score, and G-mean. Differential metabolite screening based on *p*-values and fold change, together with SHAP-based feature prioritization across multiple machine learning models, was integrated to identify candidate metabolites. The selected metabolites, together with BMI, were then incorporated into a multivariable logistic regression model to construct a nomogram.

**Results:**

No significant differences in age or sex were observed between groups, whereas BMI was higher in the CaOx group (*p* < 0.05). Twenty differential metabolites were identified (|log₂FC| > log₂ [1.5], p_adj < 0.05). SHAP analysis consistently highlighted seven metabolites across algorithms. Integration of differential and SHAP-based selection yielded five key metabolites, which, combined with BMI, produced a model with an AUC of 0.8439 (95% CI: 0.8071–0.8806), outperforming any single indicator.

**Conclusion:**

This study integrates morning urine organic acid and inorganic ion profiling with machine learning to establish a predictive model for CaOx nephrolithiasis. Five urinary metabolites were identified as potential biomarkers, providing a convenient tool for risk assessment and new insights into CaOx stone pathogenesis.

## 1 Introduction

Urolithiasis is one of the most common diseases in urology, with a global prevalence ranging from 1.5 to 18%, and its incidence continues to rise (1). Long-term urolithiasis can lead to urinary tract obstruction and infection, and in severe cases, may progress to end-stage renal disease (ESRD) (2). Based on compositional analysis, CaOx stones represent the most prevalent type of urinary calculi, accounting for approximately 65.9% of all stones (3). In recent years, urolithiasis has been increasingly recognized as a systemic metabolic disorder rather than a purely local disease of the urinary tract (4). Studies have demonstrated a close relationship between urolithiasis and human metabolic alterations (5).

At present, both the optimal timing and methodology for metabolic evaluation of urolithiasis remain controversial. Conventional assessment based on 24-h urine analysis of common metabolites provides only limited predictive power for stone formation and recurrence (4, 6, 7). From a mechanistic perspective, the formation of CaOx stones has mainly been investigated through the concentrations of urinary lithogenic ions and the balance between inhibitors and promoters of crystallization. The predominant theories include the Randall’s plaque hypothesis, the supersaturation crystallization hypothesis, and the inhibitor deficiency hypothesis (8–10). Accumulating evidence indicates that the formation of Randall’s plaques is closely related to oxidative stress, inflammation, and local osteogenic transformation, while certain metabolites, such as succinate, can influence the expression of related genes and affect both plaque formation and CaOx crystal adhesion (11). According to the supersaturation crystallization hypothesis, alterations in specific urinary metabolites may lead to precipitation of solutes such as calcium oxalate, thereby initiating crystal formation (12). The inhibitor deficiency hypothesis proposes that specific urinary metabolites, such as citrate, can inhibit crystal nucleation, aggregation, and growth. Collectively, these findings suggest that urinary lithogenic ions and metabolites play essential roles in both renal stone formation and stone-induced renal injury.

Organic acids (OAs) are a class of low-molecular-weight metabolites widely present in urine. These compounds sensitively reflect metabolic fluctuations under physiological or pathological conditions and have been considered potential biomarkers of great importance in urolithiasis research (11). Although untargeted metabolomics studies have provided valuable insights into the association between various organic acids and urinary stone disease, targeted metabolomic analyses focusing specifically on urinary OAs in CaOx nephrolithiasis remain lacking (13). Previous human studies have provided important but still incomplete evidence regarding urinary metabolomics in calcium oxalate stone disease. In a 2019 study, Wang et al. (5)applied an untargeted UPLC-Q-TOF/MS metabolomic fingerprinting approach to morning urine samples from 36 adults with CaOx urolithiasis and 36 healthy controls, and identified 18 discriminatory metabolites mainly related to caffeine, phenylalanine, galactose, and tyrosine metabolism. The reported AUCs of individual metabolites ranged from 0.615 to 0.894, with hippuric acid showing the highest diagnostic performance. However, that study was primarily exploratory, focused on untargeted metabolite discovery, and was further limited by a relatively small sample size, which may restrict the robustness and generalizability of its findings. In addition, it did not integrate urinary ionic composition, clinical variables, or multivariable predictive modeling into a clinically applicable diagnostic framework. More recently, Zhu et al. (14)developed a nomogram for predicting calcium oxalate stones using retrospective clinical and urinary ionic data, and identified sex, age, cystatin C, triglycerides, ionized calcium, and citrate as predictive factors, with an AUC/C-index of 0.772. Nevertheless, that model was designed to distinguish CaOx stones from other stone compositions in patients already diagnosed with urolithiasis, rather than to evaluate metabolic differences between CaOx stone formers and non-stone controls, and it did not include urinary organic acid profiling. Therefore, compared with previous reports, the present study was designed to combine targeted profiling of urinary organic acids and inorganic ions with machine learning-based modeling, with the aim of identifying robust metabolic biomarkers and establishing a more comprehensive and clinically translatable risk prediction strategy for CaOx nephrolithiasis. Therefore, in this study, urine samples and clinical data were collected from patients with CaOx stones and non-stone controls. Using metabolomic detection and analytical methods, we established a urinary metabolomic fingerprint, comprehensively compared differences in urinary organic acids and ions between groups, and identified urinary metabolic biomarkers predictive of CaOx stone formation. Based on these biomarkers and clinical variables, we developed a CaOx stone risk prediction model. This study provides a novel, noninvasive metabolic assessment strategy for the diagnosis of CaOx nephrolithiasis and offers potential targets for understanding stone-associated renal injury and developing precision prevention approaches-findings of considerable significance for disease diagnosis and prediction.

## 2 Materials and methods

### 2.1 Study population

Between February 2024 and May 2025, a total of 232 patients diagnosed with CaOx urolithiasis at Beijing Tsinghua Changgung Hospital were enrolled as the stone group, and 238 individuals without urinary stones during the same period were included as the non-stone control group. All participants were fully informed of the study purpose and procedures, and written informed consent was obtained from each subject prior to participation. All procedures conducted in this study were in accordance with the ethical standards of the institutional research committee and complied with the Declaration of Helsinki, as revised in 2013. The study protocol was reviewed and approved by the Medical Ethics Committee of Beijing Tsinghua Changgung Hospital (Approval No. 25545-0-02).

### 2.2 Inclusion and exclusion criteria

#### 2.2.1 Inclusion criteria

Stone group: (1) age between 18 and 75 years, no restriction on sex; (2) diagnosis of urolithiasis confirmed by ultrasonography, kidney–ureter-bladder (KUB) radiography, or CT urography (CTU); (3) postoperative stone composition analysis confirming calcium oxalate stones or stones containing more than 75% CaOx; (4) good compliance and ability to provide morning urine samples as required; and (5) voluntary participation with informed consent signed.

Non-stone group: (1) age between 18 and 75 years, no restriction on sex; (2) no history of renal calculi and no evidence of urinary stones detected by ultrasonography within the past 3 months.

#### 2.2.2 Exclusion criteria

(1) urinary stones caused by structural or congenital abnormalities of the urinary tract, such as renal malformations or ureteral strictures; (2) stones secondary to urinary tract infections or drug-induced lithiasis; (3) stones with defined metabolic etiologies, including renal tubular acidosis, primary hyperoxaluria, or primary hyperparathyroidism; (4) pregnancy.

### 2.3 Sample collection

Urine samples were collected according to previously established procedures for 24-h and morning urine collection. Under the guidance of clinical staff, participants provided 40 mL of fasting morning urine, which was immediately stored at −20 °C for short-term preservation and analyzed within 2 weeks. All patients in the stone group underwent surgical treatment, and stone specimens were collected and analyzed by infrared spectroscopy to confirm CaOx composition.

### 2.4 Metabolite detection

#### 2.4.1 Urinary organic acids

Urinary organic acids were analyzed using gas chromatography–mass spectrometry (GC–MS) with a commercial derivatization kit (Shenzhen Aone Medical Laboratory Co., Ltd.), following the manufacturer’s protocol. Briefly, 1 mL of urine was treated with urease to remove urea, followed by the addition of an internal standard mixture (heptadecanoic acid as the quantitative internal standard, and tropic acid and tetracosane as process control standards). After oximation and acidification, organic acids were extracted with ethyl acetate, evaporated under nitrogen, and derivatized with BSTFA + 1% TMCS. GC–MS analysis was performed on an A-Clin 2040NX system equipped with a DB-5 capillary column (30 m × 0.25 mm × 1 μm). The injector temperature was set at 280 °C. The oven temperature program was as follows: 100 °C for 4 min, increased at 4 °C/min to 280 °C and held for 11 min. Electron ionization was performed at 70 eV in full-scan mode (m/z 50–500). A total of 132 urinary organic acids were detected. Citric acid was quantified using an external calibration curve, while other metabolites were relatively quantified by calculating the peak area ratios to heptadecanoic acid.

#### 2.4.2 Urinary inorganic ions

Urinary inorganic ions, including sodium, ammonium, potassium, magnesium, calcium, chloride, sulfate, phosphate, and oxalate, were measured using an ion chromatography system (A-Clin 360, Shenzhen AIM Tech Co., Ltd., China) equipped with a Dionex IonPac™ CS12A analytical column (4 × 250 mm). Urine samples were centrifuged at 10,000 rpm for 10 min, filtered, diluted with ultrapure water, and purified using a solid-phase extraction column prior to analysis. Processed samples were then transferred to ion chromatography vials and analyzed using an automated sampler.

#### 2.4.3 Urinary creatinine, urea nitrogen, uric acid, and pH

Urinary creatinine and pH were measured using an ACR-300 creatinine analyzer (Lifotronic, China) and a calibrated pH meter, respectively, according to the manufacturers’ instructions. Urinary urea nitrogen and uric acid were determined by enzymatic assays using commercially available kits (Abbott, United States). Uric acid was quantified using the uricase method, and urea nitrogen was measured using the urease-glutamate dehydrogenase method.

### 2.5 Quality control and data processing

All metabolite levels were normalized to urinary creatinine and expressed as arbitrary units per gram creatinine (A.U./g Cr) to minimize the influence of urine dilution in spot morning urine samples. In urinary metabolomics, normalization is generally required because metabolite concentrations vary with urine volume and hydration status, and creatinine correction is one of the most commonly used approaches for this purpose. As urinary creatinine is widely used as a practical surrogate of urine concentration under relatively stable renal conditions, creatinine normalization was applied in this study to improve inter-individual comparability and reduce concentration-related bias in subsequent differential and predictive analyses.

Urinary metabolomic data were preprocessed prior to statistical analysis. Missing values were observed in only two variables, urea and uric acid. For urea, 4 values were missing in the control group (4/238) and 5 in the CaOx group (5/232); for uric acid, 9 values were missing in the control group (9/238) and 9 in the CaOx group (9/232). Given the low overall degree of missingness, mean substitution was applied to preserve the complete dataset for statistical comparison and model development. This approach was considered unlikely to materially distort the original data structure while retaining all samples for downstream analyses.

Analytical quality control was performed to monitor batch-to-batch consistency. As this study employed a targeted urinary metabolomics strategy, pooled biological QC samples were not used. Instead, standard QC samples containing eight target compounds were prepared in ultrapure water at two concentration levels (high and low). All QC samples were aliquoted and stored at −20 °C to avoid repeated freeze–thaw cycles, and were thawed slowly at 4–8 °C prior to analysis. Evaluation across five consecutive independent analytical batches, with four technical replicates in each batch, showed that the inter-batch coefficients of variation (CVs) for all eight target compounds were below 5%, indicating good analytical stability and reproducibility of the platform. In addition, internal standards were used as process controls to assess extraction and derivatization efficiency.

### 2.6 Feature selection and model construction

For predictive modeling, the dataset was first randomly divided into training and testing sets at an 8:2 ratio. Hyperparameter tuning and model optimization were performed exclusively within the training set using 10-fold cross-validation, while the testing set was held out for independent performance evaluation. All feature-selection procedures were also conducted exclusively within the training set to avoid data leakage.

Feature selection was performed using an integrated two-step strategy. First, differential metabolite analysis was conducted within the training set to identify significantly altered metabolites between calcium oxalate stone formers and non-stone formers. Metabolites with |log₂ fold change| > log₂ (1.5) and adjusted *p* < 0.05 were considered significantly different. Second, SHAP-based feature selection was performed independently within the training set using each of the four machine learning algorithms, including logistic regression (LR), random forest (RF), support vector machine (SVM), and extreme gradient boosting (XGBoost). For each algorithm, the top 20 metabolites ranked by SHAP importance were extracted, and the intersection across the four algorithms was defined as the set of consistently important machine learning-derived features. The overlap between these shared SHAP-selected metabolites and the differential metabolites identified in the first step was then used to generate the final candidate biomarker set for subsequent regression analyses.

The candidate metabolites obtained from the integrated screening strategy were further evaluated by univariable logistic regression within the training set. Variables with statistical significance were then entered into a multivariable logistic regression model together with the selected clinical variable(s) to construct the final prediction model and nomogram. Feature scaling was performed using *Z*-score normalization based exclusively on the training set, and the same parameters were applied to the testing set (Supplementary Figure S1). Model performance was assessed using the receiver operating characteristic (ROC) curve, area under the ROC curve (AUC), accuracy, sensitivity, and specificity. All statistical analyses and machine learning procedures were conducted using R software (version 4.4) and Python (version 3.8).

### 2.7 Statistical analysis

Continuous variables were expressed as median (interquartile range), and categorical variables were presented as counts and percentages. Group comparisons for continuous variables, including metabolite abundance comparisons between calcium oxalate stone formers and non-stone formers, were performed using the Wilcoxon rank-sum test (also referred to as the Mann–Whitney *U* test), while categorical variables were compared using the chi-square test.

## 3 Results

### 3.1 Demographic characteristics of the study population

The demographic characteristics of patients with calcium oxalate (CaOx) nephrolithiasis and healthy controls are summarized in Table 1. There were no significant differences between the two groups in terms of sex distribution or age (both *p* > 0.05). However, body mass index (BMI) was significantly higher in the CaOx stone-former group compared with the control group (25.0 vs. 24.4 kg/m^2^, *p* = 0.026). This finding is consistent with previous studies reporting a positive association between increased BMI and the risk of calcium oxalate stone formation (4, 15–17).

**Table 1:** Demographic characteristics of patients with calcium oxalate nephrolithiasis and non-stone-forming controls.

Indicators	Overall (*N* = 470^1^)	Control (*N* = 238^1^)	Stone (*N* = 232^1^)	*p*-value^2^
BMI (kg/m^2^)	24.7 [22.7, 27.0]	24.4 [22.5, 26.6]	25.0 [23.0, 27.4]	0.026
Man (%)	321 (68%)	161 (68%)	160 (69%)	0.835
Age (years)	56.0 [41.0, 66.0]	56.5 [37.0, 68.0]	56.0 [44.0, 64.0]	0.654

^1^Median [Q1, Q3]; *n* (%). ^2^Wilcoxon rank-sum test; Pearson’s chi-squared test.

### 3.2 Differential analysis of urinary metabolites between calcium oxalate stone formers and healthy controls

**Figure 1:**
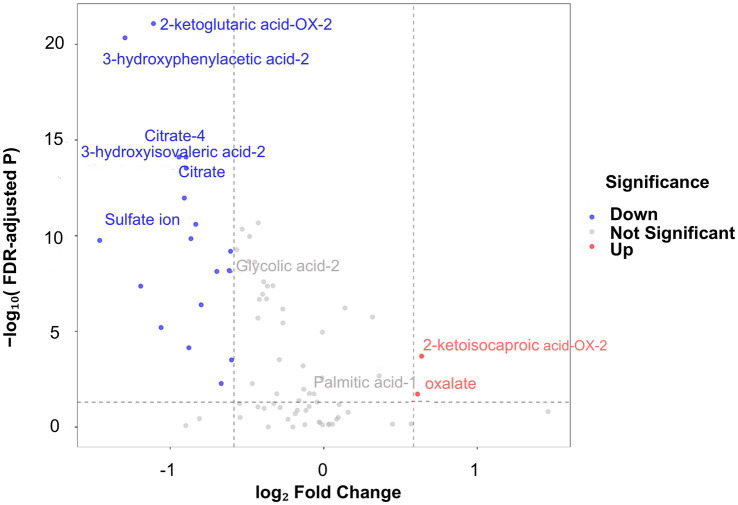
Volcano plot of differential urinary metabolites between calcium oxalate stone formers and healthy controls. The volcano plot illustrates differential analysis of urinary organic acids and inorganic ions between calcium oxalate (CaOx) stone formers and healthy controls. The x-axis represents log₂ fold change (log₂FC), and the y-axis represents −log₁₀ of the FDR-adjusted *p* value. Metabolites with log₂FC > 0 were increased in the stone-former group, whereas those with log₂FC < 0 were decreased. Vertical dashed lines indicate the fold-change threshold of |log₂FC| > log₂(1.5), and the horizontal dashed line indicates the significance threshold of adjusted *p* < 0.05. Red dots represent significantly upregulated metabolites, blue dots represent significantly downregulated metabolites, and gray dots represent metabolites without significant differences.

To investigate differences in urinary metabolite profiles between calcium oxalate stone formers and healthy controls, a differential metabolomic analysis was performed (Supplementary Table S1). Fold change (FC) was calculated to assess the direction of metabolite alterations. Metabolites with FC > 1 (log₂FC > 0) were considered upregulated in the stone-former group, whereas those with FC < 1 (log₂FC < 0) were considered downregulated. Differential metabolites with potential biological significance were defined using the thresholds |log₂FC| > log₂ (1.5) (0.585) and p_adj < 0.05. A total of 20 significantly differential metabolites were identified. Based on these results, a volcano plot was generated to visualize intergroup differences. In the volcano plot, red dots represent metabolites that were upregulated in the urine of calcium oxalate stone formers, including 2-ketoisocaproic acid-OX-2 and oxalate. In contrast, blue dots indicate 18 metabolites that were significantly decreased in stone formers compared with healthy controls, such as 3-hydroxyphenylacetic acid-2, 2-ketoglutaric acid-OX-2, 3-hydroxyisovaleric acid-2, sulfate ion, citrate, and citrate-4 (Figure 1). These differential metabolites were subsequently compared with machine learning-derived important features identified by SHAP analysis for integrated biomarker selection.

### 3.3 Construction and validation of calcium oxalate stone diagnostic models based on multiple machine learning algorithms

**Figure 2:**
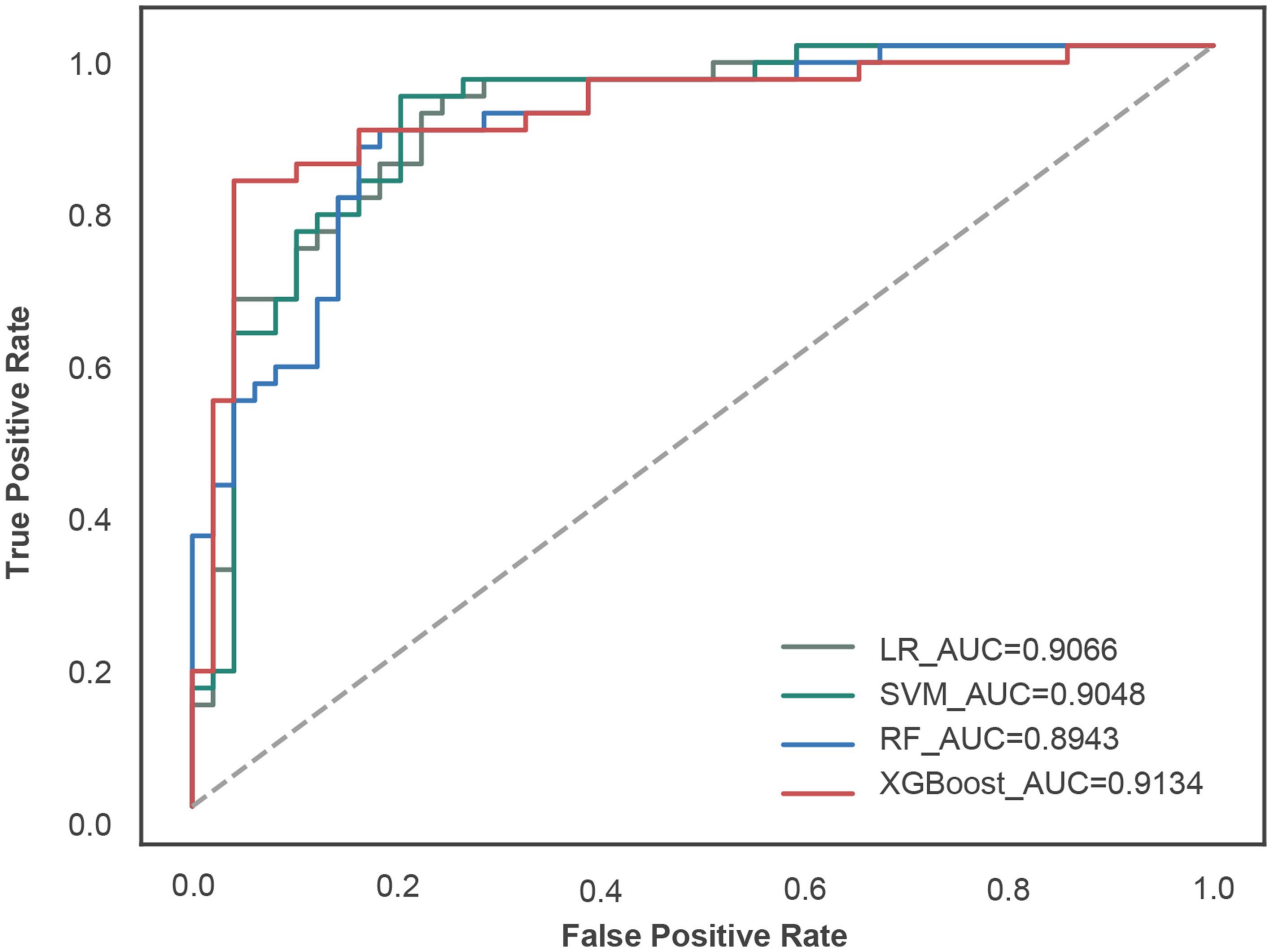
Receiver operating characteristic (ROC) curves of four machine learning models for diagnosing calcium oxalate nephrolithiasis in the independent testing set. ROC curves are shown for logistic regression (LR), support vector machine (SVM), random forest (RF), and extreme gradient boosting (XGBoost) models. The *x*-axis represents the false positive rate, and the *y*-axis represents the true positive rate. The dashed diagonal line indicates the performance of a non-informative classifier. The area under the ROC curve (AUC) for each model is shown in the legend and was used to compare discriminative performance across algorithms.

Four machine learning algorithms-logistic regression (LR), random forest (RF), support vector machine (SVM), and extreme gradient boosting (XGBoost)-were applied to construct diagnostic models for calcium oxalate nephrolithiasis (Figure 2). Hyperparameter tuning was performed using 10-fold cross-validation on the training set, yielding the optimal parameter configurations for each algorithm. The LR model used the liblinear solver with a regularization parameter of 1 and a maximum of 100 iterations. The RF model employed the Gini index as the splitting criterion, with a maximum tree depth of 25, a maximum number of features set to log₂, a minimum of one sample per leaf node, a minimum of five samples required for internal node splitting, and 50 trees. The SVM model used a linear kernel with a regularization parameter of 1 and a kernel coefficient of 3.0518 × 10^−5^. The XGBoost model was configured with a feature subsampling ratio of 1.0 per tree, a learning rate of 0.2, a maximum tree depth of 6, 100 estimators, and a subsampling ratio of 0.8. Evaluation of model performance on the independent testing set demonstrated that the XGBoost model achieved the best overall performance, with an area under the ROC curve (AUC) of 0.9134, accuracy of 0.8511, sensitivity of 0.8444, specificity of 0.8571, F1-score of 0.8444, and G-mean of 0.8508 (Table 2). Among the remaining models, LR achieved an AUC of 0.9066 with an accuracy of 0.8298, RF achieved an AUC of 0.8943 with an accuracy of 0.8298, and SVM achieved an AUC of 0.9048 with an accuracy of 0.8085. Although all four machine learning models exhibited good diagnostic performance, the XGBoost model demonstrated superior discrimination ability and overall predictive performance for calcium oxalate nephrolithiasis.

**Table 2:** Predictive performance of four machine learning algorithms (LR, RF, SVM, and XGBoost) for calcium oxalate nephrolithiasis.

Algorithms	AUC	ACC	SEN	SPE	F1-score	Gmean
RF	0. 8,943	0.8298	0.8222	0.8367	0.8222	0.8294
SVM	0.9048	0.8085	0.8222	0.7959	0.8043	0.8090
LR	0.9066	0.8298	0.8444	0.8163	0.8261	0.8303
XGB	0.9134	0.8511	0.8444	0.8571	0.8444	0.8508

RF (random forest); SVM (support vector machine); LR (logistic regression); AUC (area under the curve); ACC (accuracy); SEN (sensitivity); SPE (specificity); G-mean (geometric mean).

### 3.4 SHAP-based model interpretation and feature importance analysis

**Figure 3:**
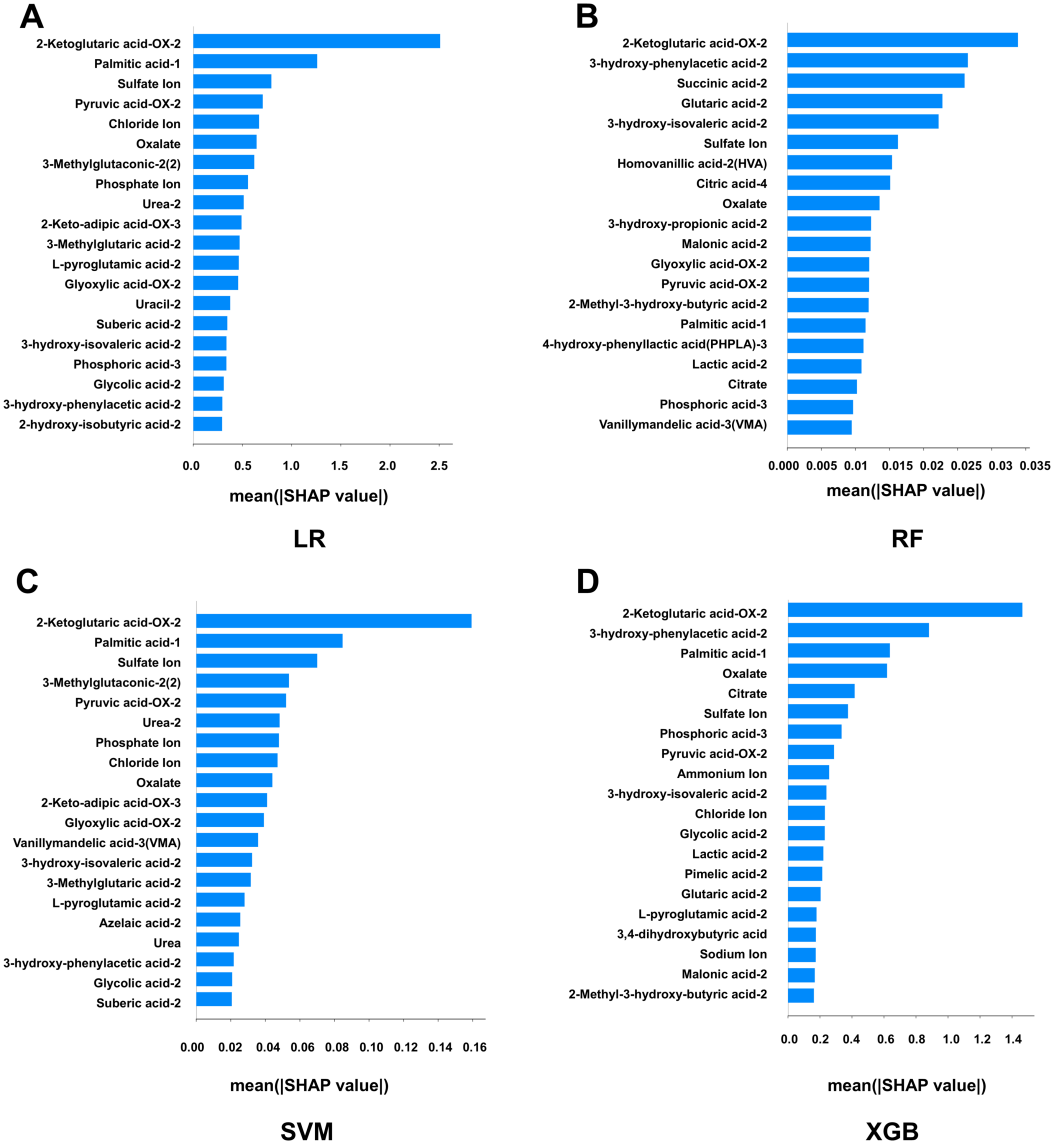
SHAP-based global feature importance of four machine learning models for calcium oxalate nephrolithiasis. **(A)** Top 20 metabolites ranked by SHAP values in the logistic regression (LR) model. **(B)** Top 20 metabolites ranked by SHAP values in the random forest (RF) model. **(C)** Top 20 metabolites ranked by SHAP values in the support vector machine (SVM) model. **(D)** Top 20 metabolites ranked by SHAP values in the extreme gradient boosting (XGBoost) model. Bar plots show the global importance of metabolites in each model after optimization by 10-fold cross-validation in the training set. Metabolites are ranked from top to bottom according to decreasing mean absolute SHAP values. The *x*-axis represents the mean absolute SHAP value (Mean |SHAP|), where higher values indicate greater overall contribution of a metabolite to model predictions.

**Figure 4:**
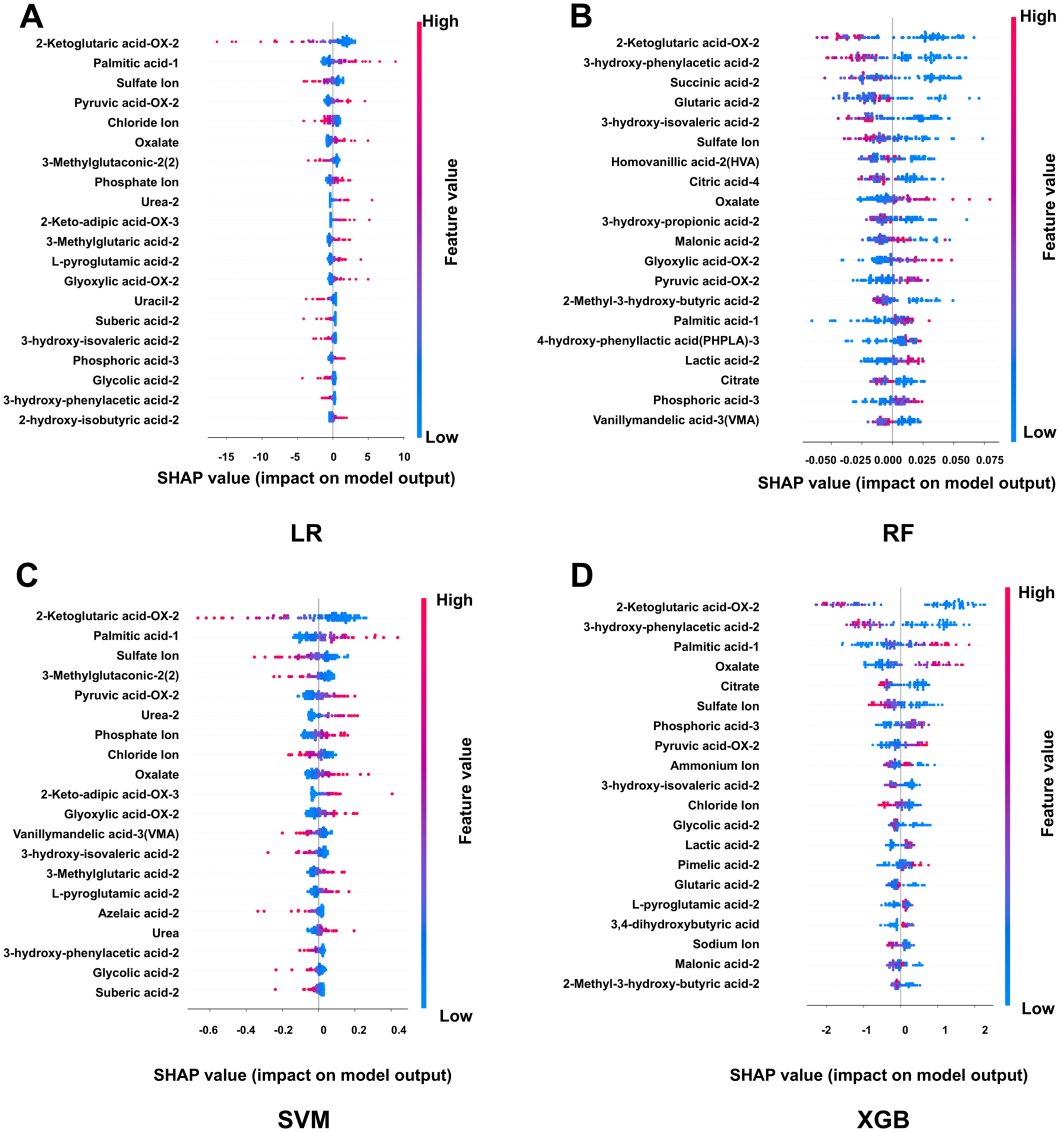
SHAP-based local interpretability of four machine learning models for calcium oxalate nephrolithiasis. **(A)** SHAP summary scatter plot of the logistic regression (LR) model. **(B)** SHAP summary scatter plot of the random forest (RF) model. **(C)** SHAP summary scatter plot of the support vector machine (SVM) model. **(D)** SHAP summary scatter plot of the extreme gradient boosting (XGBoost) model. The scatter plots illustrate local feature interpretability for the top-ranked metabolites in each model after optimization by 10-fold cross-validation in the training set. Each dot represents an individual sample. The *x*-axis indicates the SHAP value for a given feature, where positive values indicate increased contribution to stone risk prediction and negative values indicate reduced contribution. The color scale represents the relative metabolite concentration, with red indicating higher levels and blue indicating lower levels. These plots highlight the direction and magnitude of metabolite-specific effects across individual samples.

**Figure 5:**
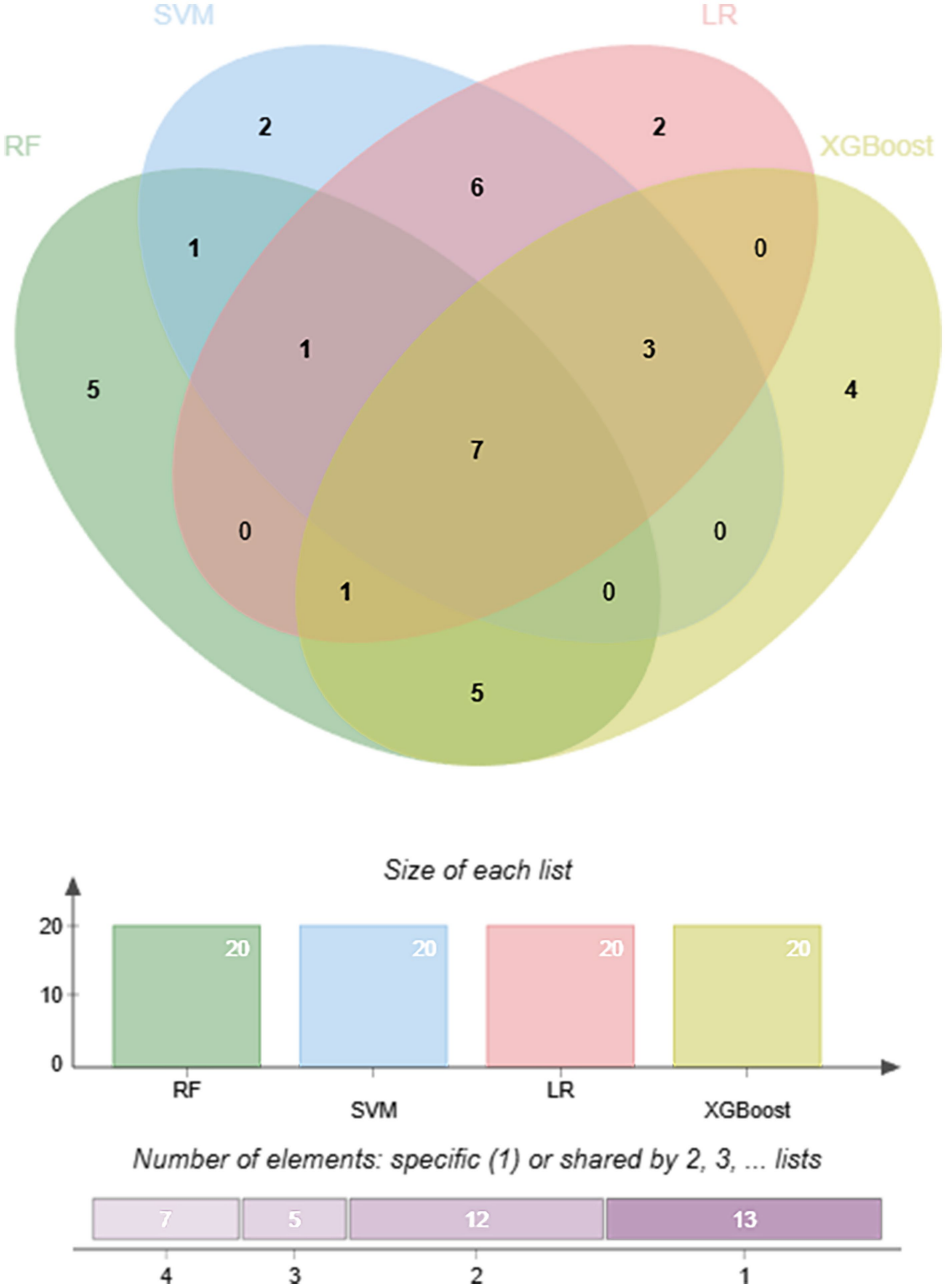
Overlap of top SHAP-ranked metabolites across four machine learning models. The Venn diagram shows the overlap among the top 20 metabolites ranked by SHAP values in the logistic regression (LR), random forest (RF), support vector machine (SVM), and extreme gradient boosting (XGBoost) models. Numbers within each region indicate the number of metabolites unique to or shared among the four models. Metabolites located in the central intersection represent features that were consistently identified as important across all four algorithms, indicating stable contributions to calcium oxalate stone prediction. The bar plots below summarize the size of each metabolite list and the number of metabolites that were specific to, or shared by, different numbers of models.

SHapley Additive exPlanations (SHAP) analysis was performed to interpret model predictions and assess feature importance by quantifying the contribution of each variable to the model output. For each of the four machine learning algorithms optimized by 10-fold cross-validation, the top 20 metabolites ranked by SHAP values were identified (Supplementary Tables S2, S3). Feature importance was visualized using bar plots and summary scatter plots for LR, RF, SVM, and XGBoost models (Figure 3). The bar plots represent global feature importance, reflecting the average contribution of each metabolite to model predictions. Among the identified metabolites, 3-hydroxyphenylacetic acid-2 and 2-ketoglutaric acid-OX-2 consistently exhibited high global importance across multiple models. Notably, 2-ketoglutaric acid-OX-2 ranked among the most influential features in all four models, suggesting a central role in distinguishing calcium oxalate stone formers from healthy controls. At the local interpretation level, SHAP summary scatter plots illustrated the direction and magnitude of each metabolite’s contribution to individual predictions (Figure 4). Each dot represents a single sample, with the *x*-axis indicating the SHAP value (positive values indicating increased stone risk and negative values indicating reduced risk), and color representing the relative metabolite concentration. This visualization highlights the heterogeneity in how metabolite levels influence model predictions across samples. To further identify robust biomarkers, metabolites with high SHAP values across all four machine learning models were integrated and analyzed using a Venn diagram (Figure 5). The intersection analysis revealed that 2-ketoglutaric acid-OX-2, 3-hydroxyphenylacetic acid-2, 3-hydroxyisovaleric acid-2, sulfate ion, palmitic acid-1, pyruvate-OX-2, and oxalate were consistently identified as important features across multiple algorithms, indicating stable and significant contributions to calcium oxalate stone prediction.

### 3.5 Construction of a multivariable clinical diagnostic nomogram for calcium oxalate nephrolithiasis

**Figure 6:**
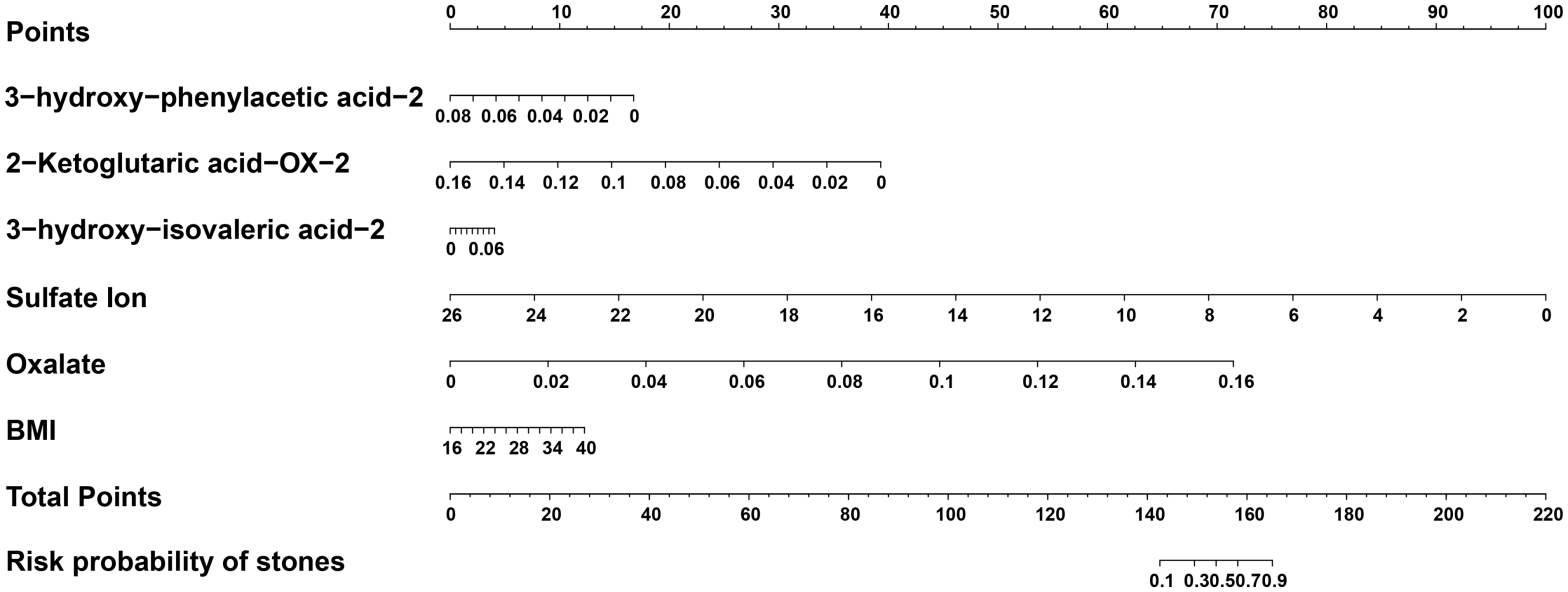
Nomogram constructed based on multivariable logistic regression analysis for the clinical diagnosis of calcium oxalate nephrolithiasis. The nomogram was constructed using six predictors: 3-hydroxyphenylacetic acid-2, 2-ketoglutaric acid-OX-2, 3-hydroxyisovaleric acid-2, sulfate ion, oxalate, and body mass index (BMI). For each variable, a corresponding point value is assigned according to its position on the scale. The total points obtained by summing the scores for all predictors correspond to the estimated probability of calcium oxalate stone formation shown on the bottom axis. Higher total scores indicate a higher predicted risk of stones.

**Figure 7:**
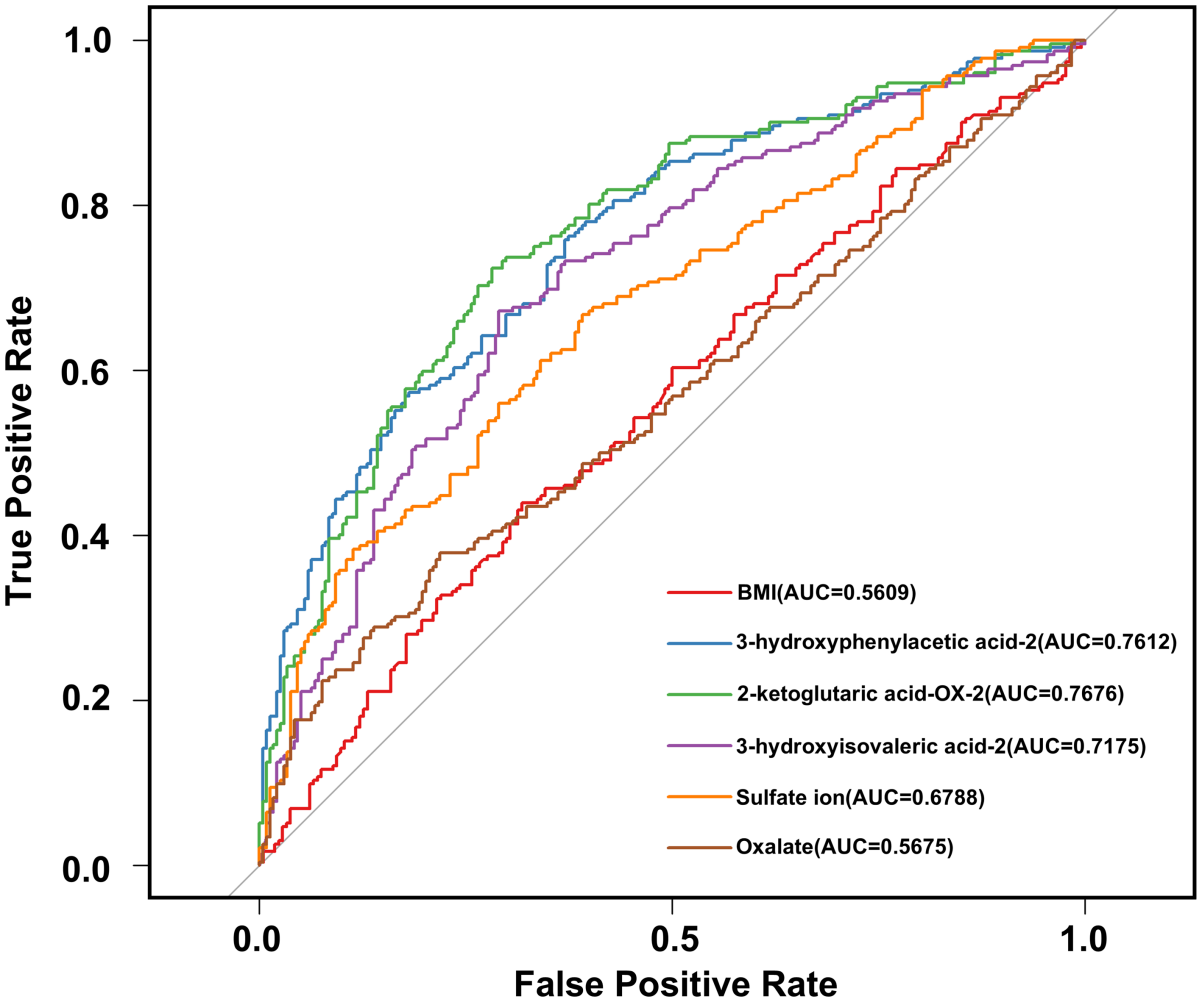
Receiver operating characteristic (ROC) curves of individual predictors for calcium oxalate nephrolithiasis. ROC curves are shown for the six individual predictors included in the multivariable model, including BMI, 3-hydroxyphenylacetic acid-2, 2-ketoglutaric acid-OX-2, 3-hydroxyisovaleric acid-2, sulfate ion, and oxalate. The *x*-axis represents the false positive rate, and the *y*-axis represents the true positive rate. The gray diagonal line indicates the performance of a non-informative classifier. The area under the ROC curve (AUC) for each predictor is shown in the legend and was used to compare the discriminatory ability of each individual variable.

By integrating differential metabolite screening with SHAP-based feature prioritization, five urinary metabolites—3-hydroxyphenylacetic acid-2, 2-ketoglutaric acid-OX-2, 3-hydroxyisovaleric acid-2, sulfate ion, and oxalate—were retained as key candidate biomarkers distinguishing stone formers from healthy controls. These five metabolites, together with BMI, were subsequently evaluated in regression analyses. Univariable logistic regression analysis identified six predictors significantly associated with calcium oxalate stone formation (Table 3). Four metabolites showed protective associations (odds ratio [OR] < 1), whereas increased BMI and oxalate levels were associated with elevated stone risk (OR > 1). Specifically, 3-hydroxyphenylacetic acid-2 (OR = 0.33, 95% CI: 0.23–0.47; *p* < 0.001), 2-ketoglutaric acid-OX-2 (OR = 0.31, 95% CI: 0.21–0.43; *p* < 0.001), 3-hydroxyisovaleric acid-2 (OR = 0.50, 95% CI: 0.36–0.66; *p* < 0.001), and sulfate ion (OR = 0.33, 95% CI: 0.23–0.48; *p* < 0.001) were negatively associated with stone formation, suggesting potential protective effects. In contrast, higher BMI (OR = 1.23, 95% CI: 1.01–1.49; *p* = 0.034) and elevated oxalate levels (OR = 1.55, 95% CI: 1.23–2.00; *p* < 0.001) were significantly associated with increased risk of calcium oxalate nephrolithiasis. These predictors were then incorporated into a multivariable logistic regression model. Based on this model, a clinical diagnostic nomogram for calcium oxalate nephrolithiasis was developed (Figure 6) to enable individualized risk estimation. Receiver operating characteristic (ROC) curves were generated for individual predictors, and the corresponding areas under the ROC curves (AUCs) were calculated to assess their diagnostic performance. Among all individual variables, 2-ketoglutaric acid-OX-2 exhibited the highest discriminative ability, with an AUC of 0.7676 (Figure 7 and Supplementary Table S4).

**Table 3:** Univariable logistic regression analysis for predictors of calcium oxalate nephrolithiasis.

Predictors	OR	95% CI	*p*-value
BMI	1.23	1.01, 1.49	0.034
3-hydroxy-phenylacetic acid-2	0.33	0.23, 0.47	<0.001
2-Ketoglutaric acid-OX-2	0.31	0.21, 0.43	<0.001
3-hydroxy-isovaleric acid-2	0.50	0.36, 0.66	<0.001
Sulfate Ion	0.33	0.23, 0.48	<0.001
Oxalate	1.55	1.23, 2.00	<0.001

OR (odds ratio).

### 3.6 Performance validation of the multivariable clinical prediction model

**Figure 8:**
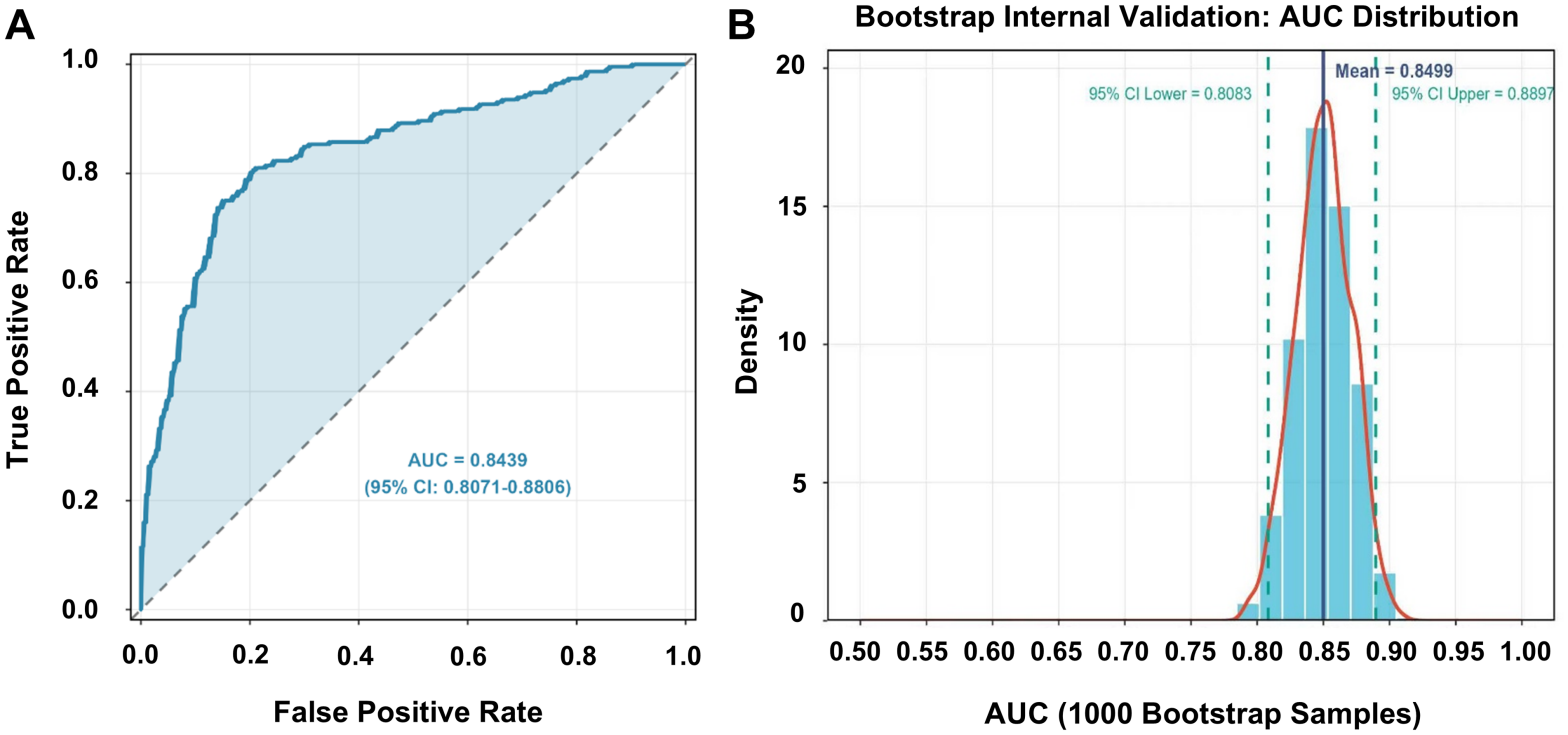
Discrimination performance and internal validation of the multivariable clinical prediction model for calcium oxalate nephrolithiasis. **(A)** Receiver operating characteristic (ROC) curve of the multivariable logistic regression model. The *x*-axis represents the false positive rate, and the *y*-axis represents the true positive rate. The dashed diagonal line indicates the performance of a non-informative classifier. The area under the ROC curve (AUC) and its 95% confidence interval are shown in the plot. **(B)** Distribution of AUC values obtained from 1,000 bootstrap samples for internal validation of the multivariable model. The histogram and density curve illustrate the distribution of bootstrap-derived AUC values. The solid vertical line indicates the mean bootstrap AUC, and the dashed vertical lines indicate the 95% confidence interval.

**Figure 9:**
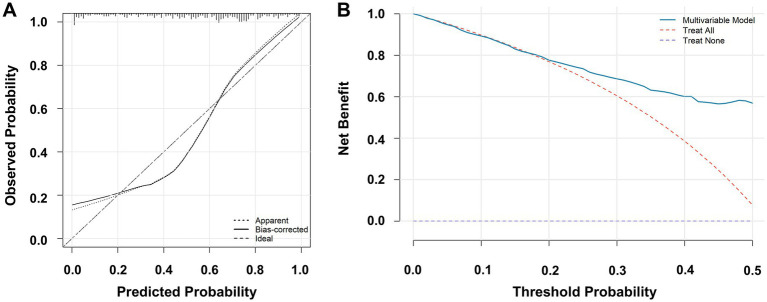
Calibration and decision curve analysis of the multivariable clinical prediction model for calcium oxalate nephrolithiasis. **(A)** Calibration curve of the multivariable logistic regression model. The *x*-axis represents the predicted probability, and the *y*-axis represents the observed probability. The dotted line indicates the apparent calibration curve, the solid line indicates the bias-corrected calibration curve, and the dashed line represents the ideal reference line. **(B)** Decision curve analysis (DCA) of the multivariable logistic regression model. The *x*-axis represents the threshold probability, and the *y*-axis represents the net benefit. The solid blue line indicates the multivariable model, the red dashed line indicates the strategy of treating all patients, and the blue dashed line indicates the strategy of treating no patients.

The multivariable logistic regression model was further subjected to a comprehensive performance evaluation to assess its clinical applicability and reliability. Model performance was evaluated across three key dimensions: discrimination, calibration, and overall accuracy. Discrimination ability, defined as the model’s capacity to distinguish calcium oxalate stone formers from healthy controls, was quantified using the AUC. An AUC greater than 0.8 is generally considered indicative of good discriminative performance. The proposed model achieved an AUC of 0.8439 (95% CI: 0.8071–0.8806), demonstrating excellent discrimination (Figure 8A). This performance was significantly superior to that of any single predictor alone, such as oxalate (AUC = 0.5675). To address potential overfitting, internal validation was performed using bootstrap resampling (1,000 iterations). The bootstrap-corrected AUC was 0.8499 (95% CI: 0.8083–0.8897), closely consistent with the original estimate, indicating good model stability and a low risk of overfitting (Figure 8B). Calibration, reflecting the agreement between predicted and observed probabilities, was evaluated using calibration curves. A well-calibrated model is essential for reliable individualized risk prediction. The calibration slope was 1 and the intercept was 0, indicating excellent agreement between predicted and observed risks. The mean absolute error was 0.064, further supporting good calibration performance (Figure 9A). Overall predictive accuracy was assessed using the Brier score, which measures the mean squared difference between predicted probabilities and observed outcomes. The Brier score of the model was 0.1668. To facilitate interpretation, a scaled Brier score was calculated as 1- (Brier score/variance of the outcome), yielding a value of 0.3317. This result indicates a 33.17% reduction in prediction error compared with a null model without predictive information. In addition, the explained variance of the model was evaluated using Nagelkerke’s *R*^2^, which was 0.407, indicating that the six predictors collectively explained 40.7% of the variability in calcium oxalate stone risk (Figure 9B). Collectively, these results demonstrate that the proposed multivariable logistic regression model exhibits excellent discrimination, calibration, and overall accuracy, with internal validation confirming its robustness, thereby providing a solid statistical foundation for potential clinical application.

## 4 Discussion

In this study, we developed a noninvasive prediction model for calcium oxalate (CaOx) nephrolithiasis by integrating morning urine organic acid and inorganic ion profiles with clinical variables and interpretable machine learning. The model achieved an AUC of 0.8439 in the independent testing set and outperformed any single clinical or metabolic indicator, supporting the value of multidimensional metabolic fingerprints in CaOx stone risk assessment. Furthermore, by combining differential metabolite screening with SHAP-based feature prioritization, we identified a set of candidate biomarkers with both statistical discriminatory ability and model-level interpretability, which may improve the clinical utility and transparency of the final model (18).

From a modeling perspective, adding urinary organic acids to conventional urinary chemistry improved the balance between sensitivity and specificity, further supporting the value of integrated metabolic profiling for stone risk stratification (19). The final nomogram translated multidimensional metabolic and clinical information into individualized risk scores, facilitating rapid risk communication in outpatient settings and supporting early intervention decisions, thus demonstrating a certain degree of clinical translational potential. Although the between-group difference in BMI was modest, BMI was retained in the final model because of its established metabolic relevance to kidney stone disease. Previous evidence has shown that overweight, obesity, and higher BMI are associated with increased urolithiasis risk, supporting the view that kidney stones are partly linked to systemic metabolic disturbance (15). Recent genetic evidence has further indicated that obesity-related traits, including BMI and waist-hip ratio, contribute to kidney stone risk and may partly mediate its association with cardiometabolic comorbidities (16). From a metabolomics perspective, BMI is also an important background variable, as metabolomic alterations in stone formers are increasingly interpreted within a broader metabolic framework (20). Moreover, BMI has been incorporated as an independent risk factor in previous predictive models for kidney stones. For example, the addition of BMI and sex to a radiomics model led to a modest improvement in predictive performance, with the AUC increasing from 0.858 to 0.860 in the training cohort and from 0.806 to 0.814 in the validation cohort. This suggests that BMI may still provide incremental clinical information even when its standalone effect is not dominant (17). Therefore, BMI was retained as a clinically relevant covariate in the final model. However, because BMI reflects only one aspect of metabolic risk and the between-group difference was relatively small, its incremental contribution to overall model discrimination should be interpreted cautiously. Further studies with larger sample sizes and external validation are needed to better define the stability and clinical utility of BMI in metabolomics-based prediction models for calcium oxalate nephrolithiasis.

From a mechanistic perspective, five key predictors were identified in our model: 3-hydroxyphenylacetic acid-2 (3-HPAA), 2-ketoglutaric acid-OX-2 (α-ketoglutarate, α-KG), 3-hydroxyisovaleric acid-2 (3-HIVA), sulfate, and oxalate. In our cohort, urinary 3-HPAA levels were markedly reduced in stone formers. As a gut microbiota-derived phenolic acid metabolite, 3-HPAA has been reported to exert antioxidant effects by suppressing ferroptosis and lipid peroxidation via the GPX4 axis, suggesting impairment of the gut-kidney metabolic antioxidant barrier in CaOx stone formers (21). Calcium oxalate crystals are known to activate the NLRP3 inflammasome in renal tubular epithelial and immune cells, triggering IL-1β/IL-18-mediated inflammatory cascades that promote crystal adhesion and tissue injury. This provides biological plausibility linking reduced 3-HPAA levels to increased oxidative and inflammatory susceptibility in stone formation (22). α-Ketoglutarate is a key intermediate of the tricarboxylic acid cycle and has been shown to suppress oxidative stress through its role in energy metabolism and to be associated with anti-aging processes (23). It also participates in metabolic-epigenetic coupling, promoting macrophage polarization toward the anti-inflammatory M2 phenotype while suppressing pro-inflammatory M1 polarization (24). In addition, *in vitro* studies have demonstrated that α-KG has a certain dissolving effect on phosphate-based stones, suggesting a protective role in maintaining the balance between crystal dissolution and inhibition. Given that Randall’s plaques-the key substrates for CaOx crystal attachment-are primarily composed of calcium phosphate deposits, reduced α-KG levels may weaken this inhibitory pathway (25). Previous studies have also linked Randall’s plaque formation to oxidative stress and immune inflammation, further supporting the role of α-KG in modulating CaOx stone formation through redox and inflammatory mechanisms (26). 3-HIVA was significantly reduced in stone formers; it is derived from leucine metabolism and is regarded as a sensitive urinary marker of mild to moderate biotin deficiency. This finding suggests a potential imbalance in vitamin-dependent energy metabolism in this population. Such metabolic states may increase susceptibility to oxidative stress and indirectly elevate stone risk by altering urinary chemistry and the urinary microenvironment, highlighting the potential importance of nutritional management in stone prevention (27). Abnormal sulfate metabolism is often associated with dietary patterns, systemic acid–base balance, and ionic interactions. High animal protein intake increases acid load, reduces urinary citrate, and elevates urinary calcium, potentially offsetting the chemical inhibitory effects of individual anions within the overall urinary milieu (28). Both modeling results and *in vitro* evidence suggest that moderate increases in urinary sulfate may reduce ionized calcium and decrease supersaturation of CaOx and calcium phosphate. However, this effect is highly dependent on accompanying urinary pH and citrate levels, indicating that clinical interventions should optimize overall urinary chemistry rather than target a single ion in isolation (29). Elevated oxalate remained the most directionally consistent risk signal in our model. Oxalate directly drives CaOx crystal nucleation, growth, and aggregation and can induce renal tubular inflammation and injury cascades, ultimately accelerating stone formation (26). Accordingly, interventions targeting oxalate burden—including dietary oxalate restriction, optimization of dietary calcium-to-oxalate ratios, and enhancement of intestinal oxalate degradation—remain critical strategies for reducing recurrence risk, particularly in high-risk populations (30).

Several methodological issues should also be considered when interpreting the present findings. First, urinary metabolite quantification relied on creatinine normalization (31). In the present study, several measures were taken to mitigate this potential bias, including the absence of significant differences in age and sex between groups, the exclusion of patients with defined metabolic causes of stones, and the use of fasting morning urine samples to reduce diurnal variation. Nevertheless, the metabolite differences observed in this study should be interpreted as creatinine-adjusted relative urinary levels rather than absolute excretion rates, and some between-group differences may still have been partly affected by variation in urinary creatinine itself. Similarly, the predictive performance of the model reflects discrimination based on creatinine-adjusted metabolite patterns rather than absolute urinary metabolite output. Future studies incorporating complementary normalization strategies, such as urine specific gravity, osmolality, or 24-h urinary excretion, as well as sensitivity analyses comparing corrected and uncorrected results, would help further validate the robustness of these findings. Second, the preprocessing strategy also warrants consideration. Mean substitution is a relatively simple approach for handling missing values. Because the proportion of missing data in the present study was low, mean imputation was considered a pragmatic and conservative choice that was unlikely to substantially alter the overall data structure while preserving the complete dataset for biomarker screening and predictive modeling (32, 33). Likewise, although no formal batch-correction algorithm was applied, all samples were processed under a unified analytical workflow, and QC samples together with internal standards were used throughout the experiment to monitor analytical stability and process performance. Nevertheless, residual effects related to missing-data handling or batch-related analytical variation cannot be entirely excluded and may have influenced biomarker selection and model performance to some extent. Third, several design-related limitations should be acknowledged. A key limitation concerns the internal validation framework. Although an independent testing set, training-set-based 10-fold cross-validation, and bootstrap analysis were used, model development still relied primarily on a single random 8:2 train/test split. In a biomarker-discovery setting, this approach may provide only limited evidence for the robustness of the overall modeling pipeline and may raise concerns regarding the stability of feature selection and model performance. Accordingly, the present findings should be interpreted with appropriate caution. In future studies, we aim to increase the sample size and replace the single random partition with repeated data splitting, while combining training-set-based 10-fold cross-validation and bootstrap analysis to enable a more comprehensive assessment of model stability and to strengthen the robustness of the modeling workflow. In addition, this was a single-center study with limited external validation; therefore, multicenter prospective studies in diverse populations will be needed to further evaluate the generalizability and calibration performance of the model. Another limitation relates to the imaging method used for defining the non-stone control group. In this study, ultrasonography was used as a screening tool mainly because it is noninvasive, radiation-free, and more feasible for control enrollment in a clinical research setting (34). However, ultrasonography is less sensitive than CT for stone detection, particularly for small or asymptomatic stones, and very small calculi may therefore have been missed in some control participants. Accordingly, the possibility of limited misclassification in the non-stone group cannot be completely excluded. Future studies using more sensitive imaging strategies, such as low-dose non-contrast CT in selected settings, may further improve control-group definition.

## 5 Conclusion

In this study, we integrated multimodal urinary organic acid and inorganic ion metabolomics with machine learning to develop a non-invasive predictive model for calcium oxalate (CaOx) nephrolithiasis. Five biomarkers—3-hydroxyphenylacetic acid-2, 2-ketoglutaric acid-OX-2, 3-hydroxyisovaleric acid-2, sulfate, and oxalate—were identified as robust predictors, potentially contributing to CaOx stone formation through enhanced Randall’s plaque development, increased urinary supersaturation, and impaired crystallization inhibition. The multivariable model achieved an AUC of 0.8439, outperforming individual clinical or metabolic indicators. Owing to its simplicity, noninvasiveness, and strong predictive performance, this approach offers a quantitative tool for risk stratification and early intervention in high-risk populations. Future multicenter prospective studies and functional investigations using cellular or animal models are warranted to validate the model and clarify the underlying biological mechanisms, thereby facilitating precision medicine in urolithiasis.

## Data Availability

The raw data supporting the conclusions of this article will be made available by the authors, without undue reservation.
