# Supplementary material for: Machine learning-driven multi-omics integration of urinary organic acids and ions enables precision risk stratification for calcium oxalate nephrolithiasis
Source: Front Med (Lausanne). 2026 Apr 20;13:1808076. doi: 10.3389/fmed.2026.1808076 (PMC13135920; doi:10.3389/fmed.2026.1808076)
Supplement: Supplementary file 1 [file Supplementary_file_1.DOCX]

**Supplemental files**

**
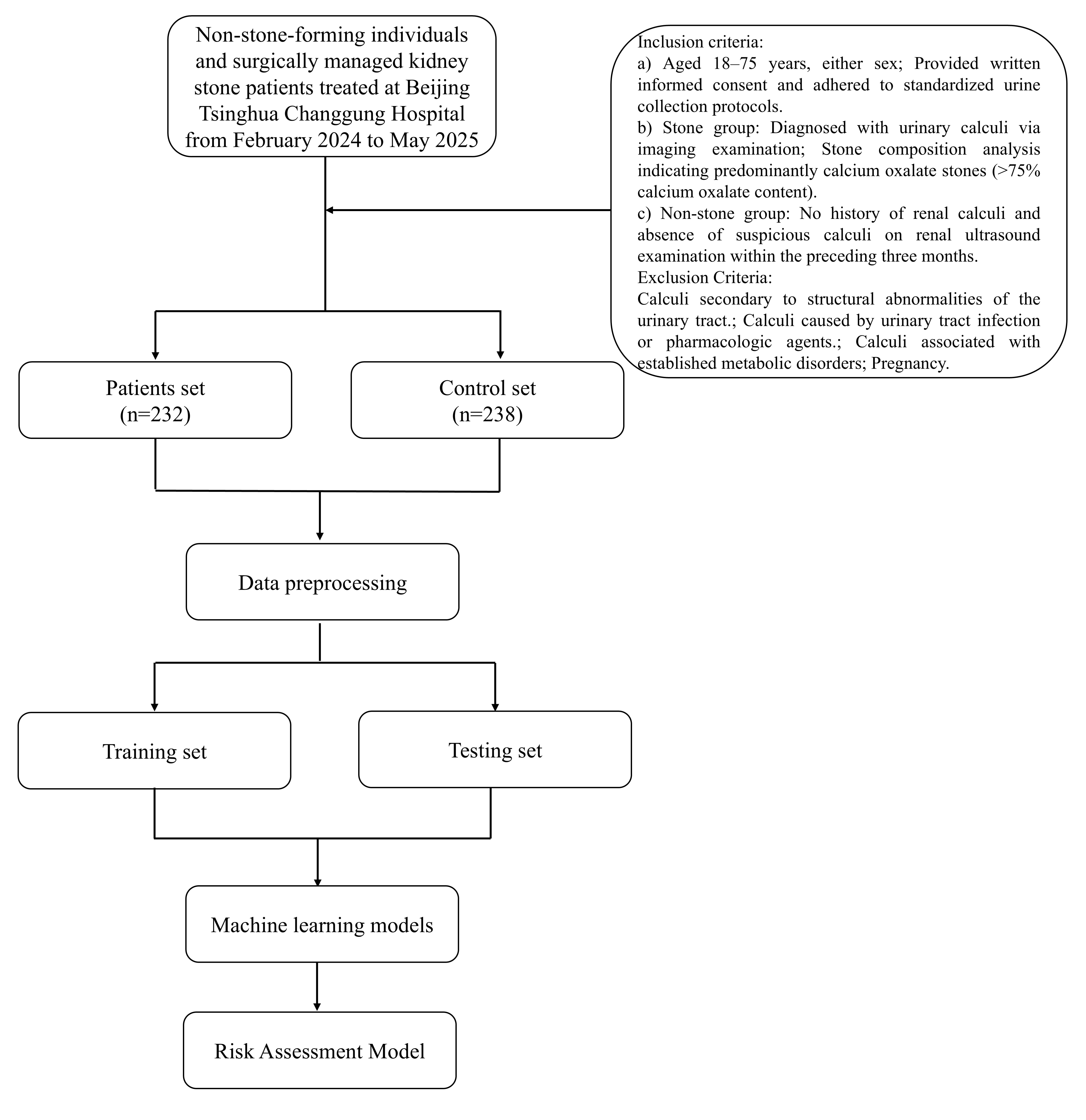
**

**Figure S1.** Risk assessment model construction flow.

**Table S1.** Differential analysis of urinary organic acids and inorganic ions between calcium oxalate stone formers and non–stone-forming controls.

| metabolite | p_val | p_adj | fc | log2fc |
| --- | --- | --- | --- | --- |
| 2-Ketoglutaric acid-OX-2 | 1.05122E-23 | 8.19948E-22 | 0.463516405 | -1.10930769 |
| 3-hydroxy-phenylacetic acid-2 | 1.16795E-22 | 4.55499E-21 | 0.407613688 | -1.2947256 |
| 3-hydroxy-isovaleric acid-2 | 3.41095E-16 | 7.78398E-15 | 0.520212334 | -0.94282749 |
| Citric acid-4 | 3.99179E-16 | 7.78398E-15 | 0.536898403 | -0.89727898 |
| Citrate | 1.9075E-15 | 2.9757E-14 | 0.537416112 | -0.89588852 |
| Succinic acid-2 | 8.28402E-14 | 1.07692E-12 | 0.532854707 | -0.90818589 |
| Homovanillic acid-2(HVA) | 1.91717E-12 | 2.13628E-11 | 0.743948061 | -0.42672619 |
| 2-Methyl-3-hydroxy-butyric acid-2 | 4.68575E-12 | 4.56861E-11 | 0.691878417 | -0.53140956 |
| Uracil-2 | 5.8967E-12 | 5.11048E-11 | 0.554890134 | -0.84972594 |
| Glutaric acid-2 | 1.39417E-11 | 1.08745E-10 | 0.715563327 | -0.48284864 |
| Sulfate Ion | 2.00725E-11 | 1.42332E-10 | 0.548934592 | -0.86529384 |
| Hippuric acid-2 | 2.71094E-11 | 1.76211E-10 | 0.363569349 | -1.45969752 |
| 3-hydroxy-propionic acid-2 | 8.93969E-11 | 5.36381E-10 | 0.675200591 | -0.56661193 |
| Potassium Ion | 1.16908E-10 | 6.51345E-10 | 0.656466464 | -0.60720678 |
| 3-Methylglutaconic-2(2) | 4.18457E-10 | 2.17597E-09 | 0.712529696 | -0.48897795 |
| Glycolic acid-2 | 4.82888E-10 | 2.35408E-09 | 0.732819519 | -0.44847016 |
| Chloride Ion | 1.44244E-09 | 6.61825E-09 | 0.652513051 | -0.61592134 |
| Sodium Ion | 1.6997E-09 | 7.36537E-09 | 0.617023846 | -0.69660185 |
| 3-Methylglutaric acid-2 | 6.23387E-09 | 2.55917E-08 | 0.76223864 | -0.39168535 |
| Vanillymandelic acid-3(VMA) | 1.04488E-08 | 4.07502E-08 | 0.794779972 | -0.33137258 |
| Phenylacetic acid-1 | 1.21578E-08 | 4.31182E-08 | 0.437485143 | -1.19269407 |
| 3-Methylglutaconic-2(1) | 1.21615E-08 | 4.31182E-08 | 0.775255151 | -0.36725689 |
| 2-hydroxymethyl-butyric acid-2 | 3.35659E-08 | 1.13832E-07 | 0.758655543 | -0.3984831 |
| Ethylmalonic acid-2 | 6.15078E-08 | 1.999E-07 | 0.772280283 | -0.37280356 |
| 2-hydroxy-isobutyric acid-2 | 6.79104E-08 | 2.11881E-07 | 0.747600969 | -0.41965966 |
| 4-hydroxy-benzoic acid-2 | 1.35773E-07 | 4.07319E-07 | 0.574813376 | -0.79883446 |
| Methylmalonic acid-2 | 2.06839E-07 | 5.97536E-07 | 1.100536399 | 0.138206862 |
| 3-methyladipic acid-2 | 2.43648E-07 | 6.78733E-07 | 0.831247769 | -0.26664953 |
| Acetylglycine-1 | 6.60853E-07 | 1.77747E-06 | 1.24604767 | 0.317359262 |
| 3-hydroxy-isobutyric acid-2 | 7.72775E-07 | 2.00922E-06 | 0.74274884 | -0.42905365 |
| Aconitic acid-3 | 1.432E-06 | 3.6031E-06 | 0.831499261 | -0.26621311 |
| 3-(3-hydroxy-phenyl)-3-hydroxy-propionic acid-3 | 2.59084E-06 | 6.31517E-06 | 0.479631069 | -1.06000298 |
| Malonic acid-2 | 4.69336E-06 | 1.10934E-05 | 0.993620718 | -0.00923284 |
| Suberic acid-2 | 3.12101E-05 | 7.15996E-05 | 0.54384433 | -0.87873434 |
| 2-Keto-isocaproic acid-OX-2 | 8.84254E-05 | 0.000197062 | 1.555573806 | 0.637446847 |
| Isocitric acid-4 | 0.00013751 | 0.000297938 | 0.818331268 | -0.28924312 |
| Glyceric acid-3 | 0.000146753 | 0.000309372 | 0.659282769 | -0.60103072 |
| Octenedioic acid-2 | 0.000306568 | 0.000629272 | 0.91060867 | -0.1350969 |
| Palmitic acid-1 | 0.001045983 | 0.002091966 | 1.284107599 | 0.360766095 |
| 3,4-dihydroxybutyric acid | 0.001378606 | 0.002688281 | 0.991578186 | -0.01220156 |
| Isobutyrylglycine-1 | 0.002843964 | 0.005281647 | 0.724176309 | -0.46558711 |
| 2-hydroxyglutaric acid-3 | 0.002840608 | 0.005281647 | 0.629321724 | -0.66813035 |
| 2-Hexenedioic acid-2 | 0.005834939 | 0.010584308 | 0.913563142 | -0.13042365 |
| 4-hydroxy-phenylacetic acid-2 | 0.009902384 | 0.017554225 | 0.936554946 | -0.09456446 |
| Urea | 0.010574203 | 0.018314912 | 0.956649888 | -0.06393707 |
| Decadienedionic acid-2 | 0.010801102 | 0.018314912 | 0.808976406 | -0.30583047 |
| Oxalate | 0.011407168 | 0.018931044 | 1.527319909 | 0.611002277 |
| Isovalerylglycine-1 | 0.025286805 | 0.041091058 | 0.894057808 | -0.16155998 |
| N-Acetylaspartic acid-2 | 0.030843545 | 0.049097887 | 0.969998535 | -0.04394553 |
| Urea-2 | 0.037054599 | 0.057805174 | 0.798089434 | -0.32537767 |
| 2-hydroxy-sebacic acid-3 | 0.038339547 | 0.058636954 | 0.68340005 | -0.54919774 |
| Lactic acid-2 | 0.044462852 | 0.066694278 | 1.071880342 | 0.100143861 |

**Table S2.** Top 20 variables ranked by SHAP values in the logistic regression and random forest models.

| LR | | RF | | |  |
| --- | --- | --- | --- | --- | --- |
| Feature | Mean shap | | Feature | Mean shap | |
| 2-Ketoglutaric acid-OX-2 | 2.5099 | | 2-Ketoglutaric acid-OX-2 | 0.0339 | |
| Palmitic acid-1 | 1.2595 | | 3-hydroxy-phenylacetic acid-2 | 0.0265 | |
| Sulfate Ion | 0.7940 | | Succinic acid-2 | 0.0260 | |
| Pyruvic acid-OX-2 | 0.7066 | | Glutaric acid-2 | 0.0228 | |
| Chloride Ion | 0.6692 | | 3-hydroxy-isovaleric acid-2 | 0.0222 | |
| Oxalate | 0.6441 | | Sulfate Ion | 0.0162 | |
| 3-Methylglutaconic-2(2) | 0.6203 | | Homovanillic acid-2(HVA) | 0.0154 | |
| Phosphate Ion | 0.5567 | | Citric acid-4 | 0.0151 | |
| Urea-2 | 0.5131 | | Oxalate | 0.0135 | |
| 2-Keto-adipic acid-OX-3 | 0.4909 | | 3-hydroxy-propionic acid-2 | 0.0123 | |
| 3-Methylglutaric acid-2 | 0.4715 | | Malonic acid-2 | 0.0122 | |
| L-pyroglutamic acid-2 | 0.4631 | | Glyoxylic acid-OX-2 | 0.0120 | |
| Glyoxylic acid-OX-2 | 0.4568 | | Pyruvic acid-OX-2 | 0.0120 | |
| Uracil-2 | 0.3755 | | 2-Methyl-3-hydroxy-butyric acid-2 | 0.0119 | |
| Suberic acid-2 | 0.3463 | | Palmitic acid-1 | 0.0115 | |
| 3-hydroxy-isovaleric acid-2 | 0.3377 | | 4-hydroxy-phenyllactic acid(PHPLA)-3 | 0.0112 | |
| Phosphoric acid-3 | 0.3369 | | Lactic acid-2 | 0.0109 | |
| Glycolic acid-2 | 0.3103 | | Citrate | 0.0102 | |
| 3-hydroxy-phenylacetic acid-2 | 0.2956 | | Phosphoric acid-3 | 0.0097 | |
| 2-hydroxy-isobutyric acid-2 | 0.2932 | | Vanillymandelic acid-3(VMA) | 0.0095 | |

**Table S3.** Top 20 variables ranked by SHAP values in the support vector machine and XGBoost models.

| SVM | | XGB | | | |
| --- | --- | --- | --- | --- | --- |
| Feature | Mean shap | | Feature | Mean shap | |
| 2-Ketoglutaric acid-OX-2 | 0.1475 | | 2-Ketoglutaric acid-OX-2 | 1.4645 | |
| Palmitic acid-1 | 0.0769 | | 3-hydroxy-phenylacetic acid-2 | 0.8813 | |
| Sulfate Ion | 0.0626 | | Palmitic acid-1 | 0.6368 | |
| 3-Methylglutaconic-2(2) | 0.0500 | | Oxalate | 0.6191 | |
| Pyruvic acid-OX-2 | 0.0479 | | Citrate | 0.4167 | |
| Urea-2 | 0.0439 | | Sulfate Ion | 0.3752 | |
| Phosphate Ion | 0.0428 | | Phosphoric acid-3 | 0.3352 | |
| Chloride Ion | 0.0418 | | Pyruvic acid-OX-2 | 0.2878 | |
| Oxalate | 0.0392 | | Ammonium Ion | 0.2574 | |
| 2-Keto-adipic acid-OX-3 | 0.0377 | | 3-hydroxy-isovaleric acid-2 | 0.2403 | |
| Glyoxylic acid-OX-2 | 0.0355 | | Chloride Ion | 0.2307 | |
| Vanillymandelic acid-3(VMA) | 0.0329 | | Glycolic acid-2 | 0.2299 | |
| 3-hydroxy-isovaleric acid-2 | 0.0298 | | Lactic acid-2 | 0.2210 | |
| 3-Methylglutaric acid-2 | 0.0295 | | Pimelic acid-2 | 0.2142 | |
| L-pyroglutamic acid-2 | 0.0258 | | Glutaric acid-2 | 0.2041 | |
| Azelaic acid-2 | 0.0229 | | L-pyroglutamic acid-2 | 0.1785 | |
| Urea | 0.0223 | | 3,4-dihydroxybutyric acid | 0.1743 | |
| 3-hydroxy-phenylacetic acid-2 | 0.0195 | | Sodium Ion | 0.1734 | |
| Glycolic acid-2 | 0.0190 | | Malonic acid-2 | 0.1675 | |
| Suberic acid-2 | 0.0186 | | 2-Methyl-3-hydroxy-butyric acid-2 | | 0.1621 |

**Table S4.** Area under the ROC curve (AUC) values of individual prognostic factors.

| Characteristic | AUC |
| --- | --- |
| BMI | 0.5609 |
| 3-hydroxy-phenylacetic acid-2 | 0.7612 |
| 2-Ketoglutaric acid-OX-2 | 0.7676 |
| 3-hydroxy-isovaleric acid-2 | 0.7175 |
| Sulfate Ion | 0.6788 |
| Oxalate | 0.5675 |
